# Supplementary material for: Phenolic Acid Profiling of Lactarius hatsudake Extracts, Anti-Cancer Function and Its Molecular Mechanisms
Source: Foods. 2022 Jun 22;11(13):1839. doi: 10.3390/foods11131839 (PMC9266154; doi:10.3390/foods11131839)
Supplement: Supplementary file 1 [file foods-11-01839-s001.zip › foods-1765482-supplementary.pdf]

Supplementary Figures

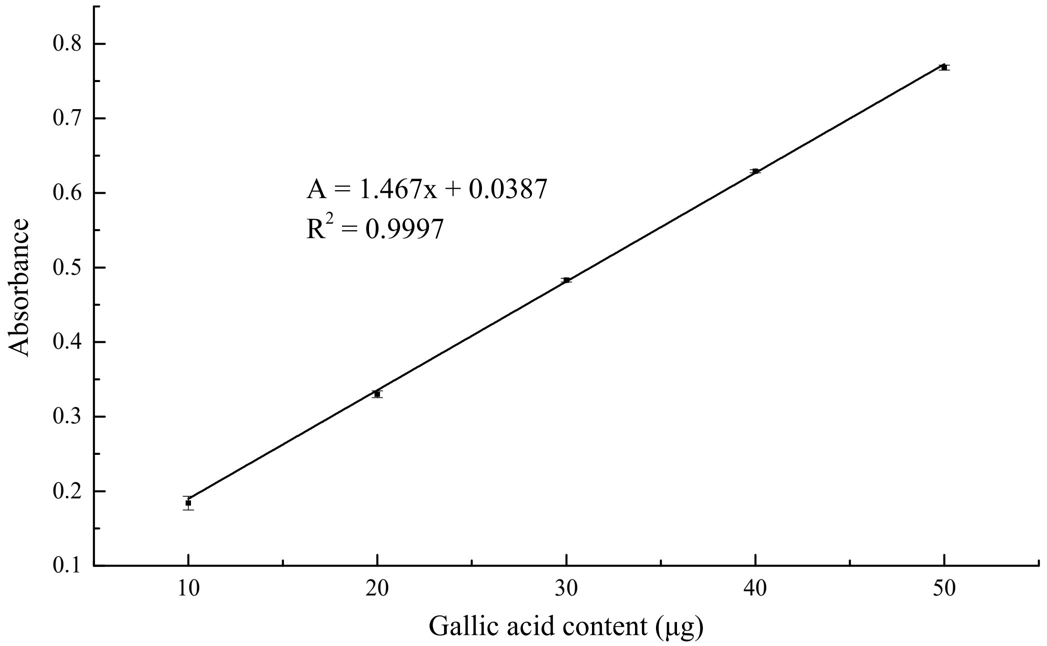

Supplementary Figure S1. Standard curve of gallic acid

The total phenolic contents with *L. hatsudake* ethanolic extracts was measured by means of the Folin-Ciocalteau assay . Gallic acid was used to calculate the standard curve. Estimation of the total phenolic acids was carried out in six duplicates (n = 6). The result was expressed as mg of gallic acid equivalents per gram of the extracts.

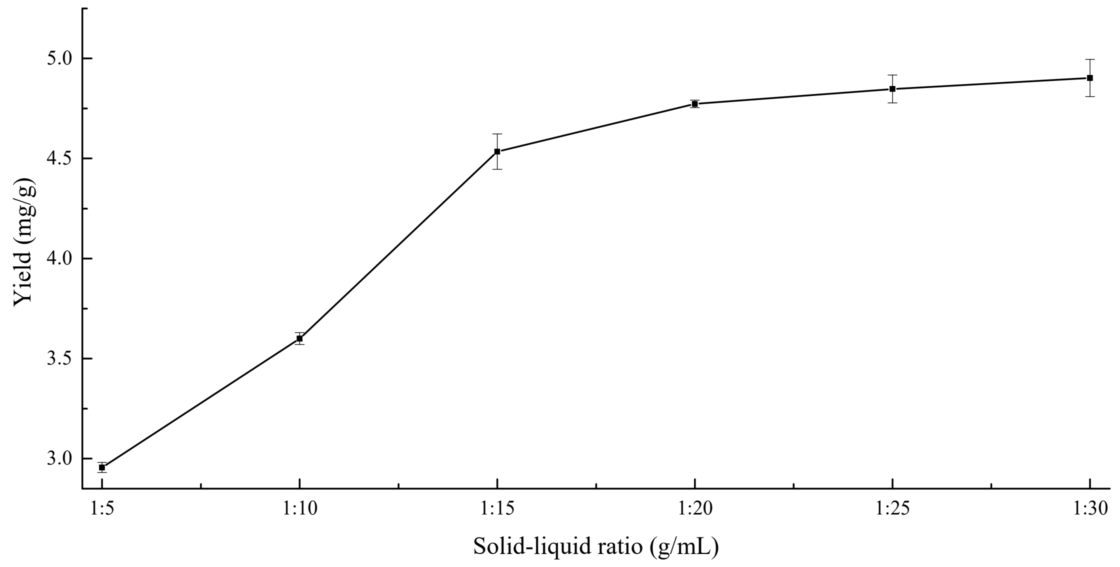

Supplementary Figure S2. Effects of solid-liquid ratio on the extraction of phenolics from *Lactarius hatsudake*.

Based on the standard curve of gallic acid, the content of phenolics in the extracts with different solid-liquid ratios (1:5, 1:10, 1:15, 1:20, 1:25, and 1:30 g/mL) was calculated respectively.

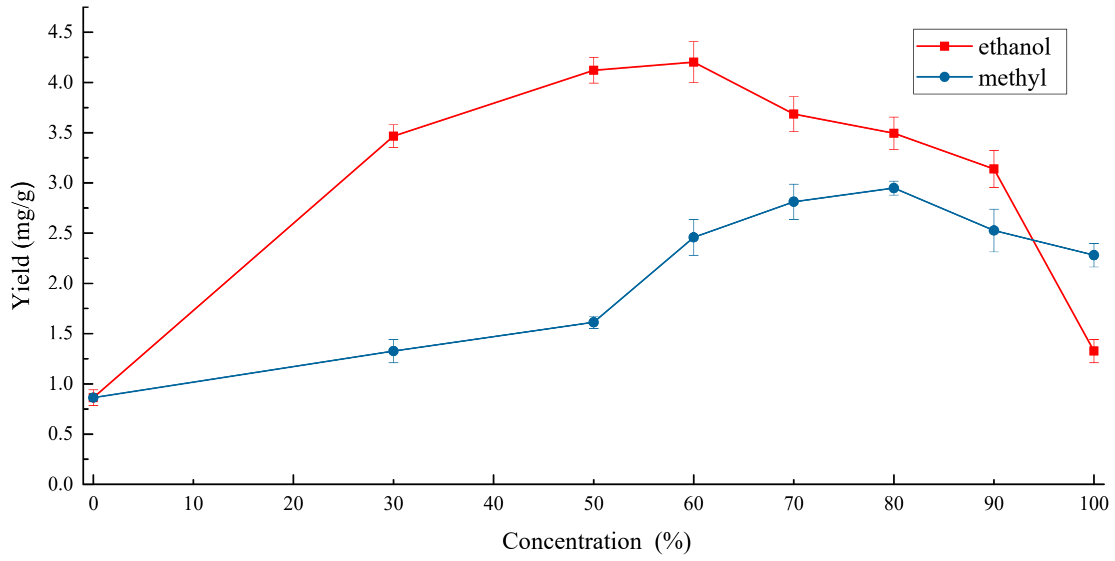

Supplementary Figure S3. Effects of solvents and concentration on the extraction of phenolics from *Lactarius hatsudake*.

Based on the standard curve of gallic acid, the content of phenolics in the extracts with different solvents and concentrations (The methanol and ethanol

concentrations were 0%, 30%, 50%, 60%, 70%, 80%, 90%, and 100%) was calculated respectively.

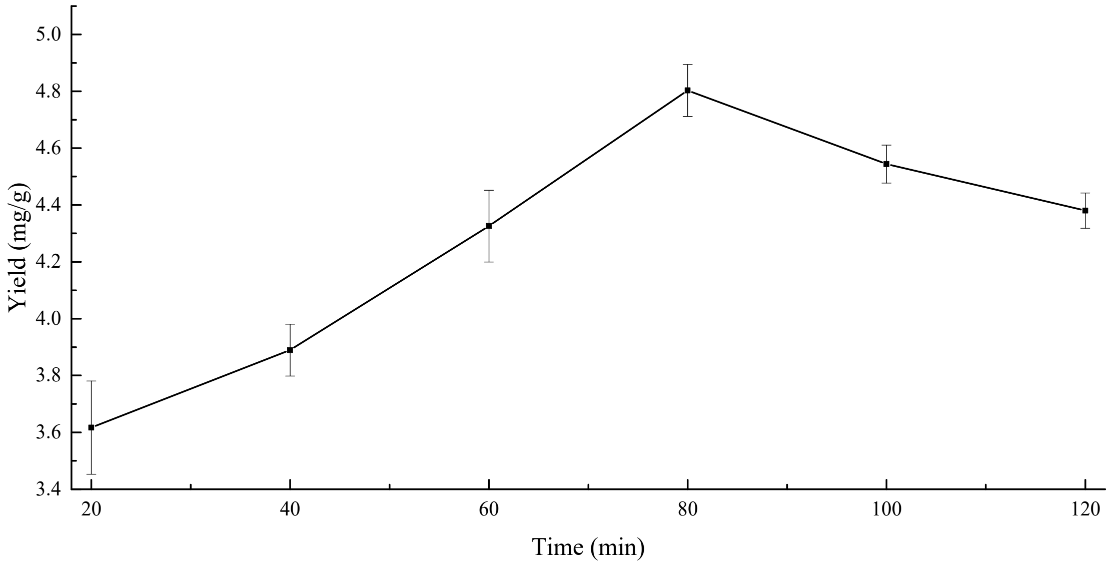

Supplementary Figure S4. Effects of extraction time on the extraction of phenolics from *Lactarius hatsudake*.

Based on the standard curve of gallic acid, the content of phenolics in the extracts with different times (20, 40, 60, 80, 100, and 120 min) was calculated respectively.
